# COVID-19 disease outcomes in patients receiving clozapine versus other antipsychotics: a national study in Qatar

**DOI:** 10.3389/fpsyt.2025.1527378

**Published:** 2025-05-08

**Authors:** Dalia Albahari, Oraib Abdalla, Shatha Mahmud Ismail Alqam, Mohammed Faisal Hamad Mohammed, Mohamed Ali Siddig Ahmed, Ovais Wadoo

**Affiliations:** ^1^ Department of Psychiatry, Hamad Medical Corporation, Doha, Qatar; ^2^ College of Medicine, Qatar University, Doha, Qatar

**Keywords:** Clozapine, COVID-19, Schizophrenia, SARS-CoV-2, antipsychotic agents, hospitalization, intensive care units, mortality

## Abstract

**Background:**

Clozapine has immunomodulatory effects that raised concerns about its potential to exacerbate severe COVID-19. This study examines whether clozapine use is associated with worse COVID-19 outcomes in patients with schizophrenia.

**Methods:**

This retrospective cohort study compared COVID-19 outcomes in SARS-CoV-2-infected patients on clozapine versus those on other antipsychotics. Primary outcomes included severe disease, hospitalization, ICU admission, and mortality. Descriptive statistics summarized the data, with categorical variables analyzed via Chi-square tests and exact Fisher test. The continuous variables were analyzed via Student’s t-test. Logistic and linear regression analyses estimated odds ratios while adjusting for confounders.

**Results:**

Thirty-three patients on clozapine (29.7%) tested positive for SARS-CoV-2 and were compared to 132 SARS-CoV-2-positive patients on non-clozapine antipsychotics. Severe infection rates did not significantly differ (clozapine: 3%, non-clozapine: 7.69%, p = 0.340), nor did hospitalization rates (clozapine: 15.1%, non-clozapine: 16.9%, p = 0.807). All clozapine patients survived, while one death (0.7%) occurred in the non-clozapine group. The mean hospital stay was similar (clozapine: 8.8 days, SD = 2.2; non-clozapine: 11.5 days, SD = 1.9; p = 0.515). Logistic regression, correcting for age, sex, vaccination status, medical comorbidities, obesity, and smoking, found no significant associations: odds ratio for severe COVID-19 = 1.9 (95% CI: 0.1–12.0, p = 0.94); odds ratio for hospitalization = 0.96 (95% CI: 0.23–3.96, p = 0.953). Linear regression of hospital stay duration yielded a β-coefficient of 4.6 (95% CI: -9.4–18.7, p = 0.471). Peri- and post-infection white blood cell and neutrophil counts were not significantly different (p = 0.4298 and p = 0.1434, respectively).

**Conclusion:**

Clozapine use was not associated with worse COVID-19 outcomes, supporting its relative safety during SARS-CoV-2 infection. These findings reassure clinicians regarding clozapine’s continued use in treatment-resistant schizophrenia. However, the small clozapine sample size limits statistical power, warranting cautious interpretation and further research.

## 1 Introduction

SARS-CoV-2, the virus responsible for COVID-19, emerged as a significant global public health issue in 2020 (1). Individuals with schizophrenia, particularly those with treatment-resistant forms who are on clozapine, face increased risks during infectious disease outbreaks like SARS-CoV-2 due to a combination of health and treatment-related vulnerabilities (2, 3). Schizophrenia is linked to various biological, social, and lifestyle factors that heighten susceptibility to infections (4). For instance, patients often have comorbidities, such as cardiovascular and metabolic disorders, which can exacerbate the severity of illnesses like COVID-19 (5–8). Additionally, cognitive and behavioral symptoms may hinder adherence to preventive measures, such as social distancing and mask-wearing, thereby increasing the risk of exposure.

Clozapine, an atypical antipsychotic designated for treatment-resistant schizophrenia, has specific immunological effects that are particularly relevant in the context of SARS-CoV-2 infection (9). In addition to its effect on multiple receptors (10–12), clozapine’s enhanced efficacy is partly explained by its immunomodulatory mechanisms, which are believed to reduce neuroinflammation that correlates with symptom control in schizophrenia, where dysfunctional immunity is a theorized pathogenesis (13–17). However, these immune effects are complex, and clozapine is also known to elevate pro-inflammatory cytokines and can cause neutropenia and, in rare cases, severe agranulocytosis (18). These hematological side effects necessitate regular monitoring, as they can impair immune function and leave patients more vulnerable to infections (19, 20). During the COVID-19 pandemic, there were concerns among clinicians that clozapine’s proinflammatory properties might exacerbate the hyperinflammatory responses seen in severe COVID-19 cases, potentially leading to poorer outcomes for these patients (3, 21). Furthermore, severe COVID-19 has been shown to affect cytochrome P450 enzymes, particularly CYP1A2, which is responsible for clozapine metabolism. This interaction may result in increased plasma levels of clozapine, raising the risk of toxicity and adverse effects during infection (22). Given these considerations, it is essential to investigate whether clozapine use is associated with worse COVID-19 outcomes in patients with schizophrenia compared to those receiving other antipsychotics. This study is the first national investigation in the Arab world to explore the relationship between clozapine use and clinical outcomes in the context of SARS-CoV-2 infection in comparison to patients on other antipsychotic medications.

## 2 Materials and methods

### 2.1 Ethics statement

The study (protocol number MRC 01-22-768) was approved by the local institutional review board.

### 2.2 Study design

This is a retrospective cohort study.

### 2.3 Setting

The Mental Health Services (MHS) of Hamad Medical Corporation is Qatar’s sole provider of public mental health services. MHS is a multidisciplinary mental health service that provides services across multiple clinical settings. MHS is the only licensed health facility in the country that prescribes clozapine. MHS’s pharmacy department keeps a registry of all the patients on clozapine and ensures adherence to the required laboratory monitoring and prescribing guidelines (23).

### 2.4 Sample and data collection

The study examined two cohorts to compare the impact of clozapine on COVID-19 clinical outcomes. The first cohort was the clozapine group, which consisted of patients who tested positive for SARS-CoV-2 while on clozapine between February 29, 2020, and November 28, 2022. The second cohort was the non-clozapine group, which consisted of individuals with severe mental illnesses (SMI), such as schizophrenia, schizoaffective disorder, and bipolar disorder, who were receiving antipsychotics other than clozapine and tested positive for SARS-CoV-2 during the same time frame to ensure comparability.

The authors retrieved eligible electronic medical records for this study through the health informatics database, employing specific search terms, including “COVID-19 positive PCR or RAT tests” and a comprehensive list of antipsychotic medications. The authors first reviewed the list of patients on clozapine to ensure adherence to inclusion criteria. Given the relatively small number of patients receiving clozapine in Qatar, all eligible individuals who met the inclusion criteria for the clozapine group were included. The authors decided to use a 1:4 ratio of clozapine to non-clozapine patients to enhance statistical power and improve the detection of meaningful differences between groups due to the smaller size of the clozapine cohort. This approach is grounded in prior research methodologies, indicating that comparisons at a ratio greater than 1:1 can yield more precise estimates and strengthen the reliability of findings, particularly in retrospective analyses with limited sample sizes (24). The non-clozapine group was randomly selected from the list of patients on other antipsychotic medications provided by the Health Informatics Department. The randomization was operated via the Microsoft Excel feature to safeguard against selection bias. All the medical charts identified after the randomization process were manually reviewed to ascertain the diagnoses of schizophrenia, schizoaffective disorder, or bipolar affective disorder. Any records of patients prescribed antipsychotic medications for conditions outside these diagnoses were excluded. The randomization process continued to replace excluded medical files until the sample size was reached. This design allowed for a relevant health risks comparison in groups that share similar diagnoses spectrum but differ in the types of antipsychotic medications. This comparison is clinically justified as these diagnoses are associated with significant functional impairment and medical comorbidities, which can increase vulnerability to infections like SARS-CoV-2.

The study evaluated several key outcomes, including:

1. Demographic and clinical characteristics of the study groups.
2. Rates of COVID-19 vaccine uptake among the two groups.
3. Rates of SARS-CoV-2 infection in the clozapine group.
4. Rates of severe COVID-19 disease in the two groups. *
5. COVID-19-related hospitalization and Intensive Care Unit (ICU) admissions in the two groups.
6. Duration of COVID-19-related hospital stays in the two groups.
7. Mortality rates among the two groups.
8. White blood cell count (WBC) and absolute neutrophil count (ANC) values for patients on clozapine at two distinct time points: during COVID-19 infection, defined as the first available WBC and ANC values within the initial two weeks following a positive SARS-CoV-2 test; and post- SARS-CoV-2 infection, defined as the first WBC and ANC values obtained between two weeks and up to six weeks after infection.

*The authors utilized the local treatment protocol for confirmed SARS-CoV-2 infections to classify disease severity. According to this protocol, severity indicators included: a) Severe pneumonia, characterized by fever or suspected respiratory infection, in conjunction with at least one of the following criteria: a respiratory rate exceeding 30 breaths per minute, severe respiratory distress, SpO2 levels below 94% on room air, or lung infiltrates exceeding 50%. b) Critical disease, defined as the occurrence of acute respiratory distress syndrome (ARDS), sepsis, septic shock, or multiple organ failure (25).

### 2.5 Data analysis

Data were analyzed using SPSS version 26. Normally distributed variables were reported as means and standard deviation (SD). Categorical variables were reported as frequencies and percentages. Chi-square test and exact Fisher test was used to compare categorical variables, and the students t-test for continuous variables. The p-value of <0.05 was considered significant. Logistic regression analysis was conducted to control for age, sex, COVID-19 vaccination, medical comorbidities, obesity and smoking status. Additionally, linear regression analysis was performed to assess the association between clinical variables and the duration of COVID-19-related hospital stay, adjusting for the same set of confounders. The results of linear regression were reported as β-coefficients with 95% confidence intervals.

## 3 Results

### 3.1 Demographic and clinical characteristics

The authors identified 33 patients with a SARS-CoV-2 positive test while taking clozapine during the study period. These 33 patients account for 29.7% of 111 patients who dispensed clozapine during the study period. The number of patients on clozapine who received antipsychotic medication augmentation was 15 (45.5%). The term “antipsychotic medications used for clozapine augmentation” refers to the addition of a second antipsychotic to clozapine therapy when clozapine alone does not lead to sufficient symptom control (**Table 1**). The authors recruited 132 non-clozapine cohort of patients who tested SARS-CoV-2 positive while taking other antipsychotic medications for the treatment of severe mental illness, as defined in the sample section. Upon comparing the clinical and demographic characteristics of the two groups (**Table 2**), the clozapine group was significantly younger than the non-clozapine group (34.9 vs. 41.4, p= 0.0088). The two groups also differed significantly in the diagnosis as the clozapine group’s most prevalent diagnosis was schizophrenia, while most of the non-clozapine group had bipolar affective disorder diagnoses (p=0.000).

**Table 1:** The antipsychotic medications used for clozapine augmentation in the clozapine group.

Name of antipsychotic medication	Frequency	Percentage
Amisulpride	4	26.67
Risperidone	3	20
Paliperidone	3	20
Aripiprazole	2	13.33
Olanzapine	1	6.67
Quetiapine	1	6.67
Zuclopenthixol decanoate	1	6.67
	15	100

**Table 2:** The demographic and clinical characteristics of the sample.

Variables	Clozapine group (N=33)	Non- clozapine group (N=132)	p-values
Female	14 (42.42%)	67 (50.76%)	0.392
Male	19 (57.58%)	65 (49.24%)	
Age in years (mean, SD)	34.9 (1.5)	41.4 (1.1)	0.0088
Nationalities			0.484
Qataris	21 (63.6%)	70 (53%)	
Non-Qatari Arabs	9 (27.2%)	36 (27.2%)	
Non-Arabs Asians	3(9%)	23 (17.4%)	
Others	0	3 (2.2%)	
Diagnosis			0.000
Schizophrenia	31(94%)	30 (23%)
Schizoaffective disorders	2 (6%)	11(8.4%)
Bipolar affective disorder	0	89 (68.4%)
Nicotine smokers	6 (20%)	25 (21%)	0.903
Chronic cardiovascular disease	0	5 (3.79%)	0.256
Diabetes mellitus	3 (8%)	31 (23.48%)	0.067
Chronic respiratory disease	5 (15.15%)	9 (6.82%)	0.124
BMI (mean, SD)	32.1(7.1)	30.8 (7.1)	0.3802

### 3.2 Vaccination uptake

The COVID-19 vaccination rate of at least a single dose of vaccine was 81.9% for the clozapine group (27/33). Only 9% of the clozapine group (3/33) contracted COVID-19 infection before the vaccination. The non-clozapine group’s vaccination rate of at least a single vaccine dose was 77.2% (102/132). The percentage of the non-clozapine group who got COVID-19 infection before vaccination was 28.4% (29/102). The differences in infection timing relative to vaccination may have influenced the observed outcomes between groups. However, due to limited availability of detailed vaccination timeline data across the full cohort, we were unable to create a three-level categorical variable distinguishing between patients vaccinated before infection, after infection, or not vaccinated at all. Instead, a binary variable indicating vaccination status (vaccinated vs. unvaccinated) was included as a covariate in regression analyses.

### 3.3 Clinical outcomes

Hospitalization Rates: Among the clozapine group, 15.1% (5/33) required hospitalization due to COVID-19. In the non-clozapine group, 16.9% (22/132) were hospitalized. The difference between groups was not statistically significant (p=0.807).

Length of Hospital Stay: The average hospital stay for COVID-19-related admissions was 8.8 days (SD 2.2) in the clozapine group and 11.5 days (SD 1.9) in the non-clozapine group, with no significant difference observed (p=0.5152).

ICU Admission Rates: No patients in the clozapine group required ICU admission, compared to 22.7% (5/22) of hospitalized patients in the non-clozapine group. However, this difference did not reach statistical significance.

Severe COVID-19 Diagnosis: Severe COVID-19 symptoms were diagnosed in 11 patients from the two groups. Only one patient on clozapine was diagnosed with a severe COVID-19 disease (9%) while the rest were from the non-clozapine group (10/11,90.91%). The proportion of patient diagnosed with severe COVID-19 disease within the clozapine group was 3% (1/33), while the proportion of patients with severe COVID-19 disease in the non-clozapine group was 7.69% (10/130). The difference was not statistically significant (p=0.340).

Mortality: No death was recorded in the clozapine group, while the non-clozapine group reported one death (0.7%), which also did not reach statistical significance (p=0.613).

No significant differences in hospitalization rates, ICU admissions, or mortality were observed between the clozapine and non-clozapine groups (**Table 3**).

**Table 3:** Comparison of the clinical outcomes between the SARS-CoV-2-positive clozapine group and the SARS-CoV-2-positive non-clozapine group.

Variables	Clozapine group (N=33)	Non- clozapine group (N=130*)	p-value
Severe COVID-19 diagnosis	1 (3%)	10 (7.69%)	0.340
COVID-19 related hospitalization ICU hospitalization	5 (15.1%) 0	22 (16.9%) 5 (22.7%)	0.807
Length of hospital days (mean, SD)	8.8 (2.2)	11.5 (1.9)	0.5152
Death	0	1(0.7%)	0.613

*1.5% missing data.

The authors conducted a logistic regression analysis for the studied outcomes to correct for age, sex, COVID-19 vaccine status, and medical comorbidities, including diabetes, cardiovascular, respiratory disease, obesity, and smoking status. The author conducted a linear regression analysis of the length of COVID-19-related hospitalization and corrected for the same potential confounders. None of the above-studied potential confounders reached statistical significance (**Tables 4**, **5**).

**Table 4:** Logistic regression analysis of severe COVID-19 disease and COVID-19 related hospitalization corrected for age, sex, COVID-19 vaccination status, diabetes, cardiovascular disease, respiratory disease, obesity, and smoking status.

Variables	Odds Ratio	95% CI	P value
Severe COVID-19 disease	1.09	0.1- 12.0	0.940
COVID-19 related hospitalization	0.96	0.23-3.96	0.953

**Table 5:** Linear regression analysis of the duration of COVID-19-related hospital stay corrected for age, sex, COVID-19 vaccination status, diabetes, cardiovascular disease, respiratory disease, obesity, and smoking status.

Variables	β-Coefficient	95% CI	P value
Duration of COVID-19-related hospital stays	4.6	-9.4-18.7	0.471

### 3.4 Laboratory findings (WBC and ANC values)

During SARS-CoV-2 infection, patients in the clozapine group had reduced mean WBC (6.5 x 10^9^/L) and ANC (3.9 x 10^9^/L) values compared to post-infection values (WBC: 6.9 x 10^9^/L, ANC: 4.5 x 10^9^/L). However, these differences were not statistically significant (**Table 6**).

**Table 6:** WBC and ANC values of patients on clozapine during and post-COVID-19 infection.

Variables	Peri-infection	Post-infection	p-value
WBC (Mean, SD)	6.5 x 10^9^/L (2.1)	6.9 x 10^9^/L (1.9)	0.4298
ANC (Mean, SD)	3.9 x 10^9^/L (2.1)	4.5 x 10^9^/L (2)	0.1434

## 4 Discussion

This national study represents the first investigation in the Arab world examining COVID-19 disease outcomes in patients with SMI receiving clozapine compared to those on other antipsychotics.

Our findings reveal important insights into the clinical implications of clozapine treatment during the COVID-19 pandemic. The clinical outcomes of the clozapine group did not show much difference from those of the non-clozapine group. Clozapine treatment was not associated with severe COVID-19 disease, frequent or lengthier COVID-19-related hospitalization, or increased mortality. Our results indicated that patients treated with clozapine exhibited hospitalization rates that were not significantly different from those on other antipsychotics. Specifically, the hospitalization rate in the clozapine group was 15.1%, compared to 16.9% in the non-clozapine cohort. These findings suggest that clozapine therapy may not exacerbate COVID-19 disease severity relative to other antipsychotics in patients with severe mental illness. Our results concur with previous studies that found no significant differences in the severity or the clinical outcomes of COVID-19 disease in patients on clozapine vs. other antipsychotic medications (26–28). This study’s findings add to the current literature on the relative safety of clozapine in SARS-CoV-2 infection and dispute the clinical translation of the hypothesized clozapine aggravation of the hyperinflammation state during severe COVID-19 disease. Clozapine’s effectiveness in treating mental illness may be partly attributed to its anti-inflammatory properties, which help modulate immune responses that are often dysregulated in psychiatric conditions. However, clozapine has a complex impact on the immune system, with both pro-inflammatory and anti-inflammatory effects, which can influence its therapeutic role and side-effect profile. Clozapine may trigger some pro-inflammatory responses, which are linked to adverse reactions like fever and inflammation in some patients, especially early in treatment. Clozapine may cause neutropenia and, in rare cases, agranulocytosis. The exact mechanism behind clozapine-induced agranulocytosis is not fully understood, but it’s thought to involve an immune-mediated response, where clozapine or its metabolites may trigger an immune attack on neutrophils, leaving patients vulnerable to infections. Clozapine’s effects on the immune system during SARS-CoV-2 infection are far more complex. The immunomodulatory profile of clozapine means that it can theoretically have both beneficial and adverse effects during a SARS-CoV-2 infection. Its anti-inflammatory properties might help reduce hyperinflammatory risks, but its pro-inflammatory tendencies and potential to lower immune cell counts can also complicate the immune response to SARS-CoV-2 infection.

Previous studies have suggested that clozapine may modulate the immune response, potentially reducing the risk of severe hyperinflammation, which is a hallmark of severe COVID-19 cases. The contrast between clozapine’s immunomodulatory effects and the potential for heightened cytokine production in patients with schizophrenia offers a complex backdrop for interpreting our results. These dynamics underscore the necessity for further exploration into how clozapine influences immune function, particularly in the context of viral infections (14, 29–33). How clozapine influences COVID-19 outcomes may vary by individual, depending on factors such as baseline immune function, dosage, and pre-existing inflammatory states. This balance makes clozapine management in COVID-19 cases complex and requires close monitoring. Given the significant benefit of continuing clozapine in this group of patients, most prescribing guidelines have advised continuing clozapine in individuals infected with SARS-CoV-2 with close monitoring for decreased white cell count and agranulocytosis (34, 35).

The rates of SARS-CoV-2 infection among clozapine-receiving patients in our sample were relatively low. This finding differs from international literature reporting increased risks of SARS-CoV-2 infection among patients on clozapine (27, 36–38). This could be explained by the limited exposure to the virus, which was directly influenced by the strict social distancing, strict lockdown regulation implemented in Qatar during the pandemic, and high vaccination rates. It could also relate to the decreased physical contact with healthcare providers due to the widespread use of telepsychiatry and the exceptional local clinical prescribing guidelines that recommended decreased laboratory testing frequencies during the COVID-19 pandemic (39–41).

Results indicated that patients on clozapine experienced only mild, transient reductions in WBC and ANC levels during SARS-CoV-2 infection. These results replicate previous literature that observed mild and transient reduction in WBC and ANC that didn’t carry a significant risk of progression to life-threatening agranulocytosis in patients infected with SARS-CoV-2 while taking clozapine. These results further strengthen the conclusion of previous studies that the transient neutropenia observed in cases of SARS-CoV-2 infections on clozapine are likely associated with the virus rather than the medications (42–45). The lack of statistically significant differences in certain expected outcomes, such as neutropenia rates between the clozapine and non-clozapine groups, could be attributed to several factors. Our sample size, particularly for the clozapine group, may have been too small to detect significant differences in rare but clinically relevant outcomes like neutropenia. Given that neutropenia is relatively uncommon, a larger cohort might be required to observe statistically significant differences in incidence, especially in the context of SARS-CoV-2 infection. Neutropenia in clozapine-treated patients can fluctuate based on various clinical factors, such as dosage adjustments, concurrent medication use, and individual susceptibility. These factors could introduce variability, potentially diluting any observable effect within a small sample. SARS-CoV-2 infection itself may cause changes in blood cell counts, including transient leukopenia and lymphopenia. This could confound our ability to isolate the effects of clozapine on neutrophil levels, as the infection may mask or mimic the hematological impacts typically associated with clozapine. Although we attempted to compare patients across both groups based on key criteria, individual health variations and other confounding factors not accounted for may have influenced the neutropenia rates. For a more robust comparison, additional variables could be controlled for, in future studies. Considering these factors, future studies with a larger and more rigorously matched cohort would likely provide a clearer picture of the relationship between clozapine use and specific immunological outcomes, such as neutropenia, especially in the context of viral infections like SARS-CoV-2.

### 4.1 Limitations

Our relatively small sample size, particularly within the clozapine group, may limit the statistical power of certain conclusions and should be considered when interpreting our findings. Additionally, the high COVID-19 vaccination rates within our sample, with most SARS-CoV-2-infected patients having been vaccinated before testing positive, may influence the generalizability of our results. While we adjusted for vaccination status using a binary variable (vaccinated vs. unvaccinated), we were unable to fully explore the effect of infection timing relative to vaccination due to limitations in our dataset. A more nuanced approach, classifying individuals as vaccinated before infection, after infection, or not vaccinated may have helped determine whether differences in infection timing masked group effects. Another important consideration is the potential confounding effect of co-prescribed medications with reported protective properties against COVID-19 severity, such as antiplatelet agents, angiotensin-converting enzyme inhibitors, angiotensin receptor blockers, statins, metformin, fluvoxamine, fluoxetine, and aripiprazole. Given that the non-clozapine group had a higher, albeit not statistically significant, prevalence of medical comorbidities, they may have been more likely to receive such medications. However, as our analysis did not reveal significant differences between groups, this potential confounder is unlikely to substantially impact on our conclusions. Finally, our study was not designed to assess the overall risk of SARS-CoV-2 infection among all individuals prescribed clozapine or other antipsychotics. The analysis was limited to those who tested positive for SARS-CoV-2 during the study period. Therefore, we did not evaluate the total number of individuals on each type of antipsychotic or the proportion who contracted COVID-19. We closed data collection once the required sample size was reached to address our predefined study objectives. While this limits insight into relative infection risk across antipsychotic groups, our study focuses specifically on comparing clinical outcomes among infected patients.

## 5 Conclusion

Despite its limitations, this study’s findings add to the current literature on the relative safety of clozapine in SARS-CoV-2 infection and dispute the clinical translation of the hypothesized clozapine aggravation of the hyperinflammation state during severe COVID-19 disease. Given the significant benefit of continuing clozapine in this group of patients, most prescribing guidelines have advised continuing clozapine in individuals infected with SARS-CoV-2 with close monitoring. Future studies should aim to include larger, more diverse populations to validate these results and further elucidate the interplay between psychiatric treatment and infectious disease outcomes. Future research should account for the potential influence of outpatient prescriptions on COVID-19 outcomes. Regional collaborations could enhance sample sizes and mitigate the challenges of conducting such studies in a relatively small population like Qatar.

## Data Availability

The raw data supporting the conclusions of this article will be made available by the authors, without undue reservation.
